# Maintenance of human chondrogenic phenotype on a dendrimer-immobilized surface for an application of cell sheet engineering

**DOI:** 10.1186/s12896-018-0426-1

**Published:** 2018-03-14

**Authors:** Sopita Wongin, Saranatra Waikakul, Pojchong Chotiyarnwong, Wanwipa Siriwatwechakul, Masahiro Kino-oka, Mee-Hae Kim, Kwanchanok Viravaidya-Pasuwat

**Affiliations:** 1 Biological Engineering Program, Faculty of Engineering, King Mongkut’s University of Technology Thonburi, Bangkok, 10140 Thailand; 2 Department of Orthopaedic Surgery, Faculty of Medicine, Siriraj Hospital, Mahidol University, Bangkok, 10700 Thailand; 3 School of Bio-Chemical Engineering and Technology, Sirindhorn International Institute of Technology, Thammasat University, Pathum Thani, 12121 Thailand; 4 Department of Biotechnology, Graduate School of Engineering, Osaka University, 2-1 Yamadaoka, Suita, Osaka, 565-0871 Japan; 5 Department of Chemical Engineering, Faculty of Engineering, King Mongkut’s University of Technology Thonburi, Bangkok, 10140 Thailand

**Keywords:** Human chondrocytes, Dendrimer surface, Morphological change, Chondrocyte sheet, Stress fiber formation, Extracellular matrix formation

## Abstract

**Background:**

Dedifferentiation of chondrocytes during cell expansion is one of the barriers in tissue construction for cartilage repair. To understand chondrocyte behavior and improve cell expansion in monolayer culture, this study investigated the effects of morphological changes and cellular aggregation on the maintenance of chondrogenic capacity by observing the expression patterns of chondrogenic (collagen type II and aggrecan) and dedifferentiation (collagen type I) markers. Primary human chondrocytes were cultured on either a polystyrene surface (PS) or a polyamidoamine dendrimer surface with a fifth-generation (G5) dendron structure to create a one-step process of cell expansion and the maintenance of chondrogenic activities prior to the construction of cell sheets.

**Results:**

During the first two passages (P0 - P2), the relative mRNA level of collagen type II decreased in all cultures, while that of collagen type I increased. Remarkably, the level of collagen type II was higher and aggrecan was retained in the chondrocytes, forming cell aggregates and showing some round-shaped cells with less production of stress fibers on the G5 surface compared to fibroblast-like chondrocytes with abundant stress fibers on the PS surface. The numbers of P2 chondrocytes on the G5 and PS surfaces were nearly the same and sufficient for construction of chondrocyte sheets using a temperature-responsive plate. Without a supporting material during cell sheet manipulation, chondrocyte sheets spontaneously detached and exhibited a honeycomb-like structure of stress fibers. Unlike the chondrocyte sheets constructed from cells on the PS surface, the chondrocyte sheets from cells on the G5 surface had higher chondrogenic activities, as evidenced by the high expression of chondrogenic markers and the low expression of dedifferentiation markers.

**Conclusions:**

The one-step process of cell expansion and maintenance of chondrogenic activity could be obtained using the G5 surface. Human chondrocyte sheets were successfully constructed with high chondrogenic activity. These findings may lead to an alternative cultivation technique for human chondrocytes that offers high clinical potential in autologous chondrocyte implantation.

**Electronic supplementary material:**

The online version of this article (10.1186/s12896-018-0426-1) contains supplementary material, which is available to authorized users.

## Background

Articular or hyaline cartilage consists of chondrocytes secreting extracellular matrix (ECM) primarily of collagen type II and aggrecan, which supports cartilage strength and resistances to mechanical stress [1, 2]. During in vitro monolayer culturing, chondrocytes often lose their original round shape with a diffuse actin network. Their shape becomes more spread with more pronounced stress fibers [3, 4]. The expressions of collagen type II and aggrecan in these cells were gradually downregulated, while that of collagen type I was upregulated, leading to the loss of chondrogenic functions known as dedifferentiated chondrocytes [5]. After transplantation of the dedifferentiated chondrocytes to cartilage defect, the regenerative tissue will eventually become fibrocartilage, which exhibits less functionality and durability compared to hyaline cartilage [6].

One of the new strategies for the treatment of cartilage defect is cell sheet technology, which allows for noninvasive cell harvesting as an intact cell sheet with their extracellular matrix [7]. This technology is applied to human articular chondrocytes to prevent the limitations of single cell injection and cartilage reconstruction using biodegradable scaffolds [7–9]. In a minipig model, chondrocyte sheets have been successfully transplanted to repair articular cartilage defects. However, some damaged areas showed fibrocartilaginous tissue with poor extracellular matrix staining [10]. Regarding the process of either cell sheet preparation or tissue-engineered cartilage, chondrocytes were passaged on a monolayer culture to generate a large number of cells, followed by three-dimensional (3D) cultivation to support the synthesis of cartilage-specific genes [11]. Unfortunately, the dedifferentiation of chondrocytes usually occurs when the cells are cultured at low cell density that promotes cellular spreading and stress fiber formation [12, 13]. Another limitation is that most cells in 3D culturing exhibit poor proliferative ability [14]. The proliferation rate of cells expanded in a 3D structure is certainly lower than that of cells grown on a 2D culture due to contact inhibition [14, 15]. Therefore, monolayer expansion of human chondrocytes in vitro has become an essential step in the process of tissue engineering and it is still a fundamental problem that needs to be addressed.

During 2D expansion of chondrocytes, many strategies including exogenous stimulation via signaling molecules have been proposed for maintaining the differentiated state of chondrocytes by introducing them into medium or onto the culture substrate [12, 16]. For instance, the exogenous transforming growth factor-β (TGF-β) can function effectively by means of arraying them in direct contact with the targets for signaling receptor on the cytoplasmic membrane [17]. However, it has not yet been proven that exogenous stimulants alone can reverse the dedifferentiated cells into chondrocytes unless the cell expansion is continuously performed in 3D carriers [12]. Alternative strategies employ intercellular signaling through regulating Rho family GTPase activity in relation to the maintenance of their phenotype expression and differentiation ability. The inhibition of actin polymerization by inactivating RhoA activity in dedifferentiated chondrocytes has been shown to change a fibroblast to round-shaped morphology and re-express chondrogenic markers [18]. As some of TGF-β signaling pathways, which target at the cell membrane, are known to crosstalk with Rho family GTPases in many cell types, it is possible that the N-cadherin-mediated cell-cell adhesions in chondrocytes are also under the control of differentiation regulators through modulating the Rho family GTPase signaling pathway [19].

In our previous studies, we designed a culture surface using a surface-immobilized polyamidoamine (PAMAM) dendrimer to regulate the cell morphology and function [20–24]. A PAMAM dendrimer has highly-branched polymers consisting of a trivalent initial core connected to primary amino groups at their terminals [22]. Following stepwise polymerization, a dendrimer layer can be generated on a PS cell culture substrate [25]. By increasing the generation of dendrimer, a large number of terminal amino groups are created, resulting in varying applications in response to cellular morphology and function [22]. In our previous work, various generations of dendrimer-immobilized surfaces were designed to retain the round shape of rabbit chondrocytes, while maintaining their chondrogenic function. Unlike the cells on a first-generation (G1) surface, the number of round-shaped cells and cellular aggregation increased with the production of collagen type II on day 3 after seeding on a fourth-generation dendrimer (G4) surface [20]. In a promising recent development, the fifth-generation dendrimer (G5) surface was designed to improve the surface property and applied to induce the aggregation of human mesenchymal stem cells (hMSCs) [21, 24, 26]. On a G5 surface, the majority of hMSCs did not only consistently form cell aggregates, but also exhibited single, round-shaped cells with temporal stretching regardless of the passage number or culture period in growth medium [26]. The analysis of cell-fate decision showed that hMSCs aggregates were also triggered to a chondrogenic fate, as shown by the localization of collagen type II around the aggregated structure [21]. We hypothesize that the changes in cell morphology and cellular aggregation on a G5 surface would have an effect on the maintenance of chondrocytes phenotype without using external growth factors and 3D cell carriers.

In this study, we employed a G5 surface as a substrate for the promotion of cellular aggregation to observe the phenotype of human chondrocytes, freshly isolated from cartilage, compared with a PS surface. Based on the findings related to the expression patterns of chondrogenic and dedifferentiation markers, the changes of cell morphology on the G5 surface and the markers may contribute to significant improvement of the chondrogenic activities before construction of chondrocyte sheets on a temperature-responsive surface.

## Methods

### Isolation of human articular chondrocytes

All procedures using human cartilage were approved by the Siriraj Institutional Review Board (COA no. Si387/2015). Human articular cartilage was obtained from osteoarthritic patients aged 64 to 70 years (3 females) who underwent total knee replacement in Siriraj Hospital, Bangkok, Thailand. All experiments were performed with osteoarthritic chondrocytes. Following enzymatic treatment, cartilage fragments were digested for 22 h at 37 °C with a digestion medium Dulbecco’s Modified Eagle Medium (DMEM; Invitrogen) containing 1.5 mg/ml collagenase II (Gibco, Grand Island, NY, USA) and 1% antibiotic-antimycotic (Invitrogen, Grand Island, NY, USA). To collect the chondrocytes, cell suspensions underwent centrifugation at 1500 rpm for 5 min. After washing twice with Hank’s Balanced Salt Solution (HBSS) containing 1% antibiotic-antimycotic, the cells were cultured in a growth medium: DMEM medium supplemented with 10% Fetal Bovine Serum (FBS; Invitrogen) and 1% antibiotic-antimycotic at 37 °C in a humidified CO_2_ incubator.

### Preparation of dendrimer-immobilized surface

Square 8-well PS plates, T75 flasks and T225 flasks (Corning Inc., NY, USA) were used to prepare G5 surface, as described previously [26]. Under sterile conditions, the PS surfaces were incubated with potassium *tert*-butoxide solution to display the hydroxyl groups for 1 h. After washing the surfaces with sterile water, they were incubated with a solution of glutaraldehyde for 1 h. The surfaces were then washed with a large amount of sterile water, followed by incubation with tris (2-aminoethyl) amine solution for 1 h and rinsing with sterile water. The excess reagent was then removed, allowing for generating a non-toxic cell culture substrate. The procedure for incubation with glutaraldehyde and tris (2-aminoethyl) amine solution was performed 5 times to obtain the G5 surface. To display terminal ligands, a d-glucose solution was added and incubated for 2 h. After that, the culture surfaces were immersed with sodium borohydride solution without the removal of d-glucose solution for 24 h and then washed with sterile water before use.

### Culture conditions for human chondrocytes

Human chondrocytes were expanded by repeated passaging to P2 chondrocytes in a growth medium on either the PS surface of T75 flasks and T225 flasks or the G5 surface of T75 flasks and T225 flasks for cell sheet construction. The culture medium was changed ever 3–4 days.

For real-time PCR and immunostaining analysis, the freshly isolated chondrocytes were either lysed for RNA extraction or cultured for repeated passaging (P0 – P2) on the uncoated square 8-well plates (Nunc, Roskilde, Denmark) or the G5 surface of square 8-well plates. In all passages, the seeding density of chondrocytes was fixed at 5 × 10^3^ cells/cm^2^. RNA extraction and cell fixation were performed on day 7 of each passage.

### Preparation of human chondrocyte sheets on a temperature – Responsive surface

After expansion of chondrocytes on either the G5 surface or PS surface, P2 chondrocytes were used for the construction of monolayer and triple-layered cell sheets. The 24-well temperature-responsive plates (CellSeed, Tokyo, Japan) were pre-coated with FBS at 37 °C for 1 day. Teflon rings (diameter, 1.1 cm) were inserted into the well plates to prevent cell attachment at the edge of the well. To form a monolayer sheet, the chondrocytes were seeded at a density of 3 × 10^5^ cells/cm^2^, and cultured at 37 °C in a humidified CO_2_ incubator for 24 h. To release a monolayer cell sheet from the culture plate, Teflon rings and the supernatant were removed, leaving only a sufficient amount of solution to cover the cell surfaces. After that, the culture plates were incubated at 20 °C for 30 min. At this temperature, the chondrocyte sheet detached itself and shrank slightly. After mild agitation with serum-free medium, the entire cell sheet with the medium was aspirated into a plastic dropper. Next, the first layer of cell sheet was transferred onto FBS-coated 35-mm tissue culture polystyrene dish and incubated at 37 °C for 30 min to allow for cell attachment. By performing these processes three times, triple-layered chondrocyte sheets were obtained. The chondrocyte sheets constructed from P2 chondrocytes on either the G5 surface or PS surface were cultured for 7 days at 37 °C in a humidified CO_2_ incubator.

### Analysis of gene expression

Total RNA extraction was carried out using the RNeasy Mini Kit (Qiagen, Hilden, Germany) according to the manufacturer’s instructions. Total RNA was reverse transcribed into complementary DNA using a PrimeScript RT Reagent Kit (Takara Bio Inc., Shiga, Japan). Quantitative real-time PCR was performed using SYBR Premix Ex Taq (Takara Bio Inc.) and a Stratagene Mx3005P (Agilent Technologies, CA, USA). The primers used in real-time PCR were shown in Table 1. The target genes were amplified by 40 amplification cycles of 95 °C for 3 s and 60 °C for 30 s. Relative expression levels were normalized to glyceraldehyde-3-phosphate dehydrogenase expression and calculated using the ${2}^{-\Delta {\mathrm{C}}_{\mathrm{t}}}$ method [27].

**Table 1:** Primer sequences used for real-time PCR

Primer ID	Primers (5’**➔**3′)	Ref.
Collagen type I-F	CAGACAAGCAACCCAAACTGAA	[37]
Collagen type I-R	TGAGAGATGAATGCAAAGGAAAAA	
Collagen type II-F	GGCAATAGCAGGTTCACGTACA	[38]
Collagen type II-R	CGATAACAGTCTTGCCCCACTT	
Aggrecan-F	AGCCTGCGCTCCAATGACT	[39]
Aggrecan-R	TAATGGAACACGATGCCTTTCA	
Glyceraldehyde-3-phosphate dehydrogenase-F	CAACGGATTTGGTCGTATTGG	[40]
Glyceraldehyde-3-phosphate dehydrogenase-R	GCCATGGGTGGAATCATATTG	

### Immunofluorescence staining

To observe the expression of stress fibers and chondrogenic markers, the chondrocytes and chondrocyte sheets were subjected to immunofluorescence staining on day 7 after seeding on a tissue culture polystyrene dish. The cells were fixed with 4% paraformaldehyde and incubated with 0.5% polyoxyethylene (10) octylphenyl ether in PBS for permeabilization. Non-specific binding protein was reduced in a Block Ace solution (Dainippon Sumitomo Pharma Co., Ltd., Osaka, Japan) for 90 min. The cells were incubated in primary antibody for human collagen type II, collagen type I, and aggrecan (Santa Cruz Biotechnology, CA, USA) at 4°C overnight. After washing with Tris-buffered saline (TBS), a solution of secondary antibody (Alexa Fluor 488 donkey anti-goat IgG, Molecular Probes, OR, USA) was added and incubated for 1 h. Another incubation was conducted for 40 min with 4′, 6-diamidino-2-phenylindole (Molecular Probes) and fluorescein-rhodamine phalloidin (Molecular Probes) to enable the visualization of nucleus and actin filaments, respectively. The images of chondrocytes were obtained using Cytell System (GE Healthcare Life Science, Pittsburg, PA). The images of chondrocyte sheets were obtained using a confocal laser scanning microscope (CLSM, model FV1000; Olympus, Tokyo, Japan).

### Statistical analysis

All experiments were performed at least three times, with data expressed as means with standard deviations. These values were used in one-way ANOVA followed by Tukey’s test to determine statistical significance (*P* < 0.05).

## Results

### Behaviors of human chondrocytes on G5 surface

**Fig. 1:**
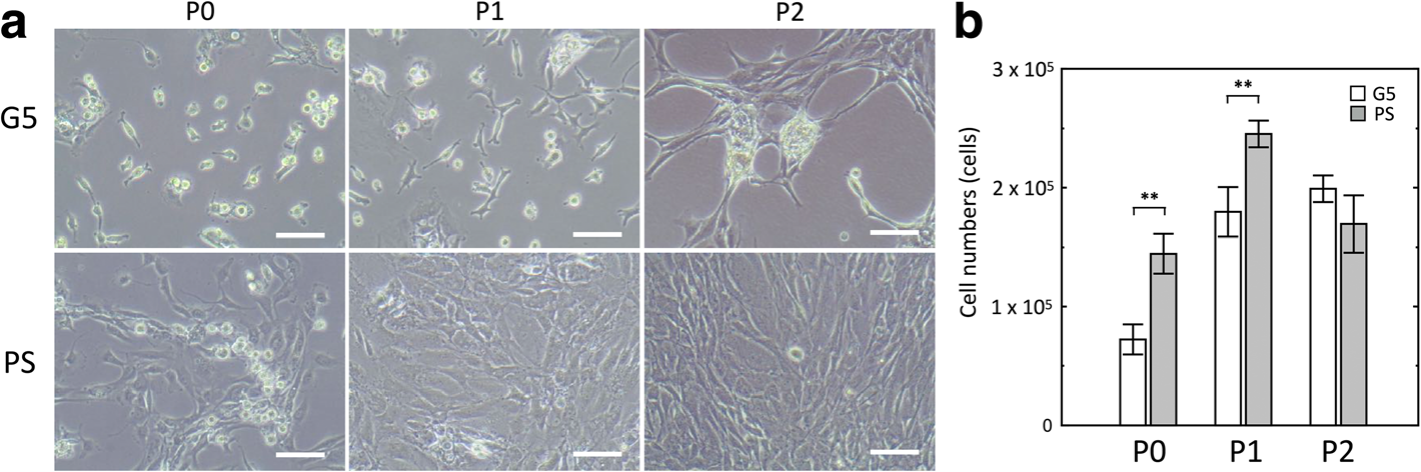
Morphology of human chondrocytes at passage 0–2 on G5 and PS surfaces on day 7 (**a**). Scale bars show 100 μm. Cell numbers after cultivation of human chondrocytes on G5 and PS surfaces on day 7 (**b**). Data represent average values with the standard deviation determined from triplicate independent experiments. Differences among the data sets were considered significant at **p < 0.01

The morphology and numbers of freshly isolated (P0) and passaged (P1 and P2) chondrocytes were investigated on the G5 and PS surfaces on day 7 after seeding. As shown in Fig. 1a, the freshly isolated chondrocytes showed a typical round shape, similar to native chondrocytes, and small cell aggregates on the G5 surface, although some cells exhibited a polygonal shape. Conversely, most of the freshly isolated cells on the PS surface showed a polygonal shape with some round-shaped cells. When the chondrocytes were passaged on the G5 surface, P1 chondrocytes did not only show the round and polygonal morphology, but also formed small cell aggregates. The dense aggregated forms of the cells were clearly observed in P2 chondrocytes, although elongated polygonal cells were also detected. On the PS surface, all P1 and P2 chondrocytes were elongated with a fibroblast-like structure and reached confluency in a monolayer manner without aggregate formation.

In this study, a large number of chondrocytes was required for cell sheet construction. During serial passage, cell proliferation was also observed. To investigate the effect of a dendrimer surface on cell proliferation, the cell numbers of P0 - P2 chondrocytes on either the G5 surface or PS surface were counted on day 7 after seeding. As shown in Fig. 1b, the cell numbers of P0 and P1 chondrocytes on the G5 surface were significantly lower than those of the chondrocytes on the PS surface. However, the cell numbers of P2 chondrocytes on both surfaces were nearly the same, even though the confluency percentage may be different. It was considered that the high number of cells was almost clumped in the dense aggregated forms of the chondrocytes on the G5 surface. These results indicated that the chemical process for preparing G5 surface did not affect cell viability of chondrocytes.

### Chondrogenic properties of human chondrocytes

**Fig. 2:**
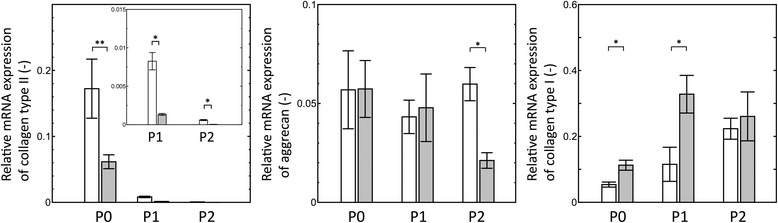
Quantitative RT-PCR analysis of collagen type II, aggrecan and collagen type I expressions in human chondrocytes cultured on G5 (open bar) and PS (closed bar) surfaces. Data represent average values with the standard deviation determined from three independent experiments and considered significant at *p < 0.05 and **p < 0.01

To observe whether the chondrogenic properties of human chondrocytes were maintained on a dendrimer-immobilized surface, the chondrogenic (collagen type II and aggrecan) and dedifferentiation (collagen type I) markers were investigated in the cells cultured on G5 and PS surfaces. As shown in Fig. 2, the mRNA level of collagen type II in the human chondrocytes on both surfaces was reduced after two serial passages. Interestingly, the collagen type II expression was significantly higher in P0 - P2 chondrocytes cultured on the G5 surface than that in the cells cultured on the PS surface. Moreover, the mRNA levels of aggrecan expression in the chondrocytes on the G5 surface were quite similar among P0 – P2 cells. However, expression in the P2 chondrocytes on the PS surface was significantly lower than in the P2 chondrocytes on the G5 surface. Regarding the dedifferentiation marker, a tendency toward a more increased mRNA level of collagen type I was detected in the passaged chondrocytes on both cultures. In the earlier passage (P0 – P1), the expression of collagen type I was significantly less in the chondrocytes on the G5 surface compared with that in the cells on the PS surface. This result indicated that the G5 surface supported the expansion of chondrocytes while maintaining their chondrogenic capacity.

**Fig. 3:**
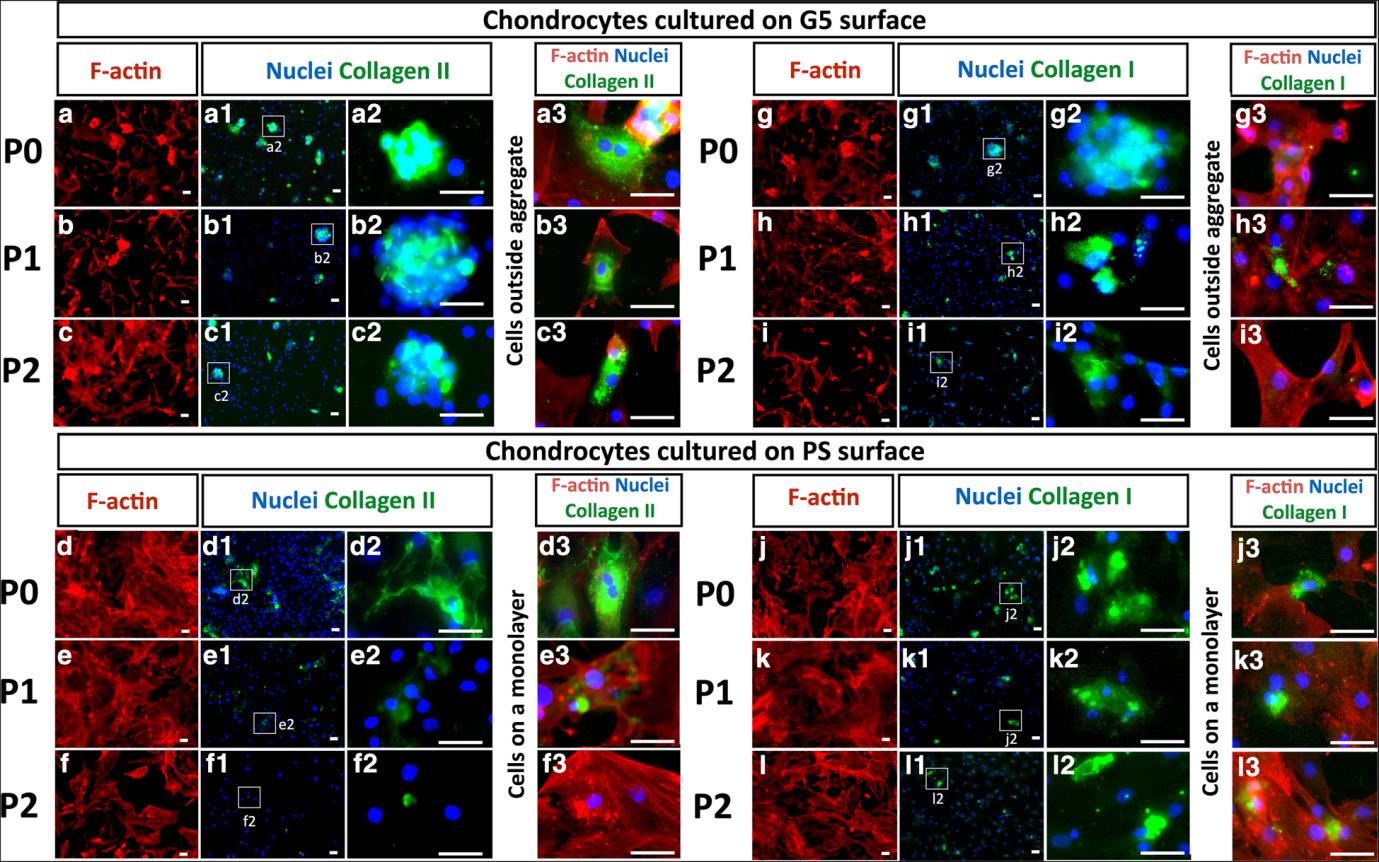
Immunofluorescence staining for collagen type II (a1-f3), collagen type I (g1-l3) (green), F-actin (a-l) (red), and nuclei (blue) in human chondrocytes at passage 0–2 on G5 and PS surfaces. Panels a2-l2 show the enlargments of white box areas in panels a1-l1, respectively. Scale bars show 100 μm

According to the formation of cell aggregates on the G5 surface, the relationship between the cell morphology and expression of chondrocyte markers was observed by immunostaining of F-actin, collagen type II and collagen type I. As shown in Fig. 3, immunostaining of F-actin in all cultures was found in most of the elongated polygonal shape of chondrocytes with thick and linear structure of stress fibers (Fig. 3a – l). However, F-actin staining was undetectable in some round-shaped cells of P0 - P2 chondrocytes on the G5 surface and P0 chondrocytes on the PS surface (Fig. 3a3 – d3). On the G5 surface, the expression of collagen type II was found mainly in the cell aggregates of P0 - P2 chondrocytes (Fig. 3a1 – c1 and a2 – c2). Moreover, the localization of collagen type II could be observed only in the cells, even in the cells outside the aggregates, which exhibited negative staining for F-actin (Fig. 3a3 – c3). On the PS surface, collagen type II staining was visualized only in P0 chondrocytes (Fig. 3d1 – d3), whereas P1 - P2 chondrocytes showed negative staining for collagen type II (Fig. 3e1 – e3 and f1 – f3). However, collagen type I staining was also detected in all cultures and its expression was particularly observed in the cells cultured on the PS surface (Fig. 3j1 - l3). Therefore, immunostaining assay revealed that cell aggregates and the chondrocytes, which exhibited negative staining for F-actin, were positive for collagen type II.

### Properties of human chondrocyte sheets

**Fig. 4:**
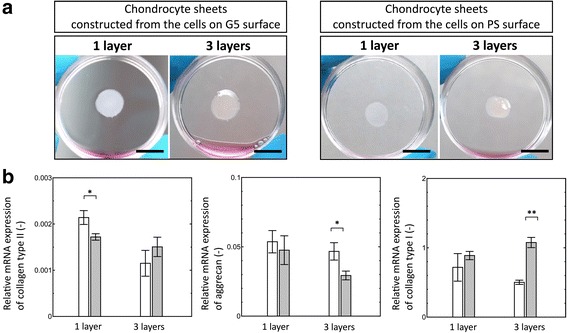
Monolayered and triple-layered chondrocyte sheets constructed from the cells on G5 and PS surfaces. Scale bars show 1 cm (**a**). Quantitative RT-PCR analysis of collagen type II, aggrecan and collagen type I expressions in human chondrocytes sheets constructed from the human chondrocytes on the G5 (open bar) and PS (close bar) surfaces (**b**). Data represent average values with the standard deviation determined from triplicate independent experiments and considered significant at *p < 0.05 and **p < 0.01

To verify whether the chondrocytes expanded on either the G5 surface or PS surface have the capacity to generate cartilage-like tissue, the expression of chondrogenic and dedifferentiated markers in the chondrocyte sheets were investigated after 7 days of culture by gene expression and immunostaining analysis. As shown in Fig. 4, a sufficient number of chondrocytes expanded on either the G5 surface or the PS surface was successfully constructed as a dense cell sheet (Fig. 4a). The relative mRNA level of collagen type II in the monolayer chondrocyte sheet, which was constructed from the cells on the G5 surface, was higher than that in the monolayer chondrocyte sheet constructed from the cells on the PS surface (Fig. 4b). However, there was no significant difference between the levels of collagen type II in the triple-layered chondrocyte sheets constructed from the cells on the G5 surface and PS surface. The mRNA expression of aggrecan was retained in the monolayer and triple-layered chondrocyte sheets constructed from the G5 surface, but its expression was reduced in triple-layered cell sheets constructed from the cells cultured on the PS surface. In all chondrocyte sheets, the marker for dedifferentiated chondrocytes, collagen type I, was also detected. The mRNA level of collagen type I was increased in the chondrocyte sheets constructed from the cells expanded on the PS surface, compared to that of the cell sheets constructed from the cells on the G5 surface. Particularly, the mRNA level of collagen type I was significantly decreased in the triple-layered chondrocyte sheets constructed from the cells on G5 surface.

**Fig. 5:**
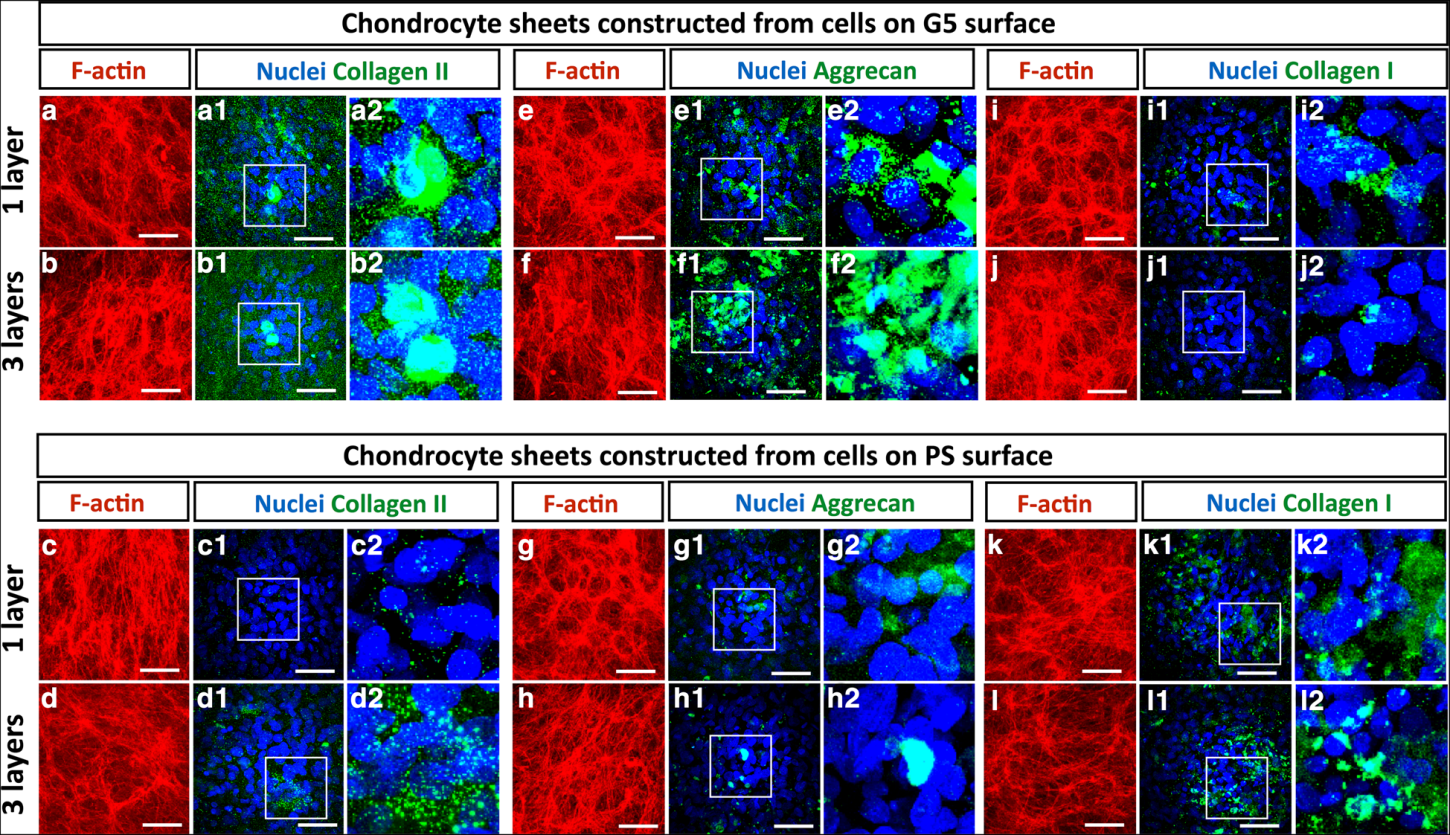
Immunofluorescence staining for collagen type II (a1-d2), aggrecan (e1-h2), collagen type I (i1-l2) (green), F-actin (a-l) (red) and nuclei (blue) in monolayer and triple-layered chondrocyte sheets constructed from chondrocytes on G5 and PS surfaces. Panels a2-l2 show the enlargements of white box areas in panels a1-l1, respectively. Scale bars show 50 μm

To visualize the overall shape and structure of the cells after cell sheet construction, F-actin in the chondrocyte sheets were stained with rhodamine phalloidin. The localization of chondrogenic and dedifferentiated markers was also investigated by immunofluorescence staining. As shown in Fig. 5, the overall shape of the cells inside the dense structure of chondrocyte sheets exhibited a round shape. The F-actin staining revealed that the cells have very few and thin stress fibers with a honeycomb-like structure throughout both the monolayer and triple-layered chondrocyte sheets (Fig. 5a - l). For immunostaining of chondrogenic markers, the positive staining of collagen type II and aggrecan was observed in all of the chondrocyte sheets. The localization of chondrogenic markers was strongly detected in the round-shaped cells inside the monolayer and triple-layered chondrocyte sheets constructed from the cells on the G5 surface (Fig. 5a1 - b2 and e1 – f2), compared to that in the cells on the PS surface (Fig. 5c1- d2 and g1 – h2). For the dedifferentiation marker, the localization of collagen type I was distributed throughout all the chondrocyte sheets (Fig. 5i1 - l2). However, staining for collagen type I was more intense in the chondrocyte sheets constructed from the cells on the PS surface (Fig. 5k1 - l2) compared with that in the cell sheets constructed from the cells on the G5 surface (Fig. 5i1 - j2). These results revealed that the chondrocyte sheets constructed from P2 chondrocytes on the G5 surface exhibited an increased capacity to retain chondrogenic activity and led to a decreased dedifferentiated marker compared to the PS surface.

## Discussion

This study demonstrated a culture strategy that facilitated the acquisition of large quantities of cells following a sequential expansion process and cell sheet engineering. A one-step process for chondrocyte expansion and maintenance of chondrogenic phenotype on a G5 surface for cell sheet construction without the addition of chondrogenic supplement was highlighted.

The key factors involved in the regulation of chondrogenic phenotype are interactions between cell-cell and cell-ECM via cadherin and integrin functions [28]. During skeletal development, condensed MSCs in the early phase of chondrogenesis frequently generate cell-cell connections via adhesion molecules such as cadherin [29]. These cells are then triggered toward a chondrogenic fate and become chondrocytes. In the later stage, ECM produced by chondrocytes plays a role in cell proliferation via integrin and other cell surface receptors before development into hypertropic chondrocytes and undergoing endochondral ossification [1, 30].

To maintain the chondrogenic stage of human chondrocytes during in vitro monolayer culture, we considered that the regulation of chondrocyte behaviors is required by increasing cell-cell adhesion and reducing cell-ECM adhesion on the substrate. From the results of morphological change, most of the chondrocytes passaged on G5 surface exhibited round-shaped morphology with cellular aggregation (Fig. 1). After serial passage, a sufficient number of cells were also obtained, potentially leading to the proposal of a one-step process for cell expansion and maintenance of chondrogenic activity, as shown in Additional file 1. On the G5 surface, cells exhibited active extension and strong contraction with unstable adhesion due to the adsorption and assembly of fibronectin matrix [23]. The changes of cell morphology and active migration led to the formation of cell aggregates during cell division and coalescence of migrating cells [23, 26]. When the culture period was extended, the dense aggregate of cells subsequently generates more cell-cell adhesion [31]. Previous work reported that the aggregation of rabbit chondrocytes, which exhibited cell-cell adhesion and expression of N-cadherin molecule, have been shown to increase the synthesis of collagen type II [20]. Consistent with the results in this study, human chondrocyte aggregates increased the potential expression of chondrogenic markers, as evidenced by the localization of collagen type II protein in the cell aggregates on the G5 surface (Fig. 3). Moreover, gene expression results support the fact that cell aggregation is a primary key in the retaining chondrocyte phenotype because the higher expression of collagen type II gene and the remaining aggrecan were found in the cells passaged on a G5 surface, compared to the monolayer cells without aggregate formation on a PS surface. These results suggest that the production of chondrogenic markers via high cadherin-mediated adhesion in the cell aggregate could preserve the chondrogenic state of human chondrocytes during culture on the G5 surface.

An increase in chondrocyte attachment to the culture substrate, as a result of integrin-mediated adhesion, was seen after a prolonged culture, followed by morphological changes to flatten-shaped cells with the production of collagen type I [32]. The results in this study reconfirmed that most of the flattened-shaped cells, which prevailed on the PS surface, showed drastic upregulation of collagen type I gene with concomitant localization mainly in the monolayer cells (Figs. 2 and 3). Chondrocytes exhibiting flattened shape via the activation of integrin-mediated adhesion undoubtedly led to the loss of chondrogenic activity. The positive staining of collagen type I was also found in some aggregates of P0 chondrocytes on both the G5 and PS surfaces. This may have resulted from the cartilage biopsies received from osteoarthritic patient, reflecting the inability of cells to support the cartilage matrix regeneration [33]. However, the expansion of chondrocytes on the G5 surface have a tendency to reduce the dedifferentiation process, as shown by the lower mRNA level of collagen type I in the P0 and P1 chondrocytes.

In addition to these changes in cell-cell and cell-ECM interactions, the authors present the evidence that some chondrocytes passaged on the G5 surface also altered the ability to produce collagen type II by changing stress fiber formation. Changes in cell morphology rely on the dynamic reorganization of stress fibers, which are formed by the interaction of actin filaments [21, 34]. We hypothesize that cellular signal, which control stress fiber formation and cytoskeletal reorganization, might be involved in the production of chondrogenic-specific phenotype. Studies have shown that RhoA signal, a member in the Rho family GTPase, is responsible for the formation of stress fibers [18, 20]. Single cells with the disappearance of stress fibers have also been shown to produce collagen type II [35]. In this study, immunofluorescence staining revealed that some passaged chondrocytes on G5 surface exhibited less staining of F-actin with the expression of collagen type II, whereas distinct long and thick stress fibers without collagen type II expression were found in the monolayer chondrocytes on the PS surface (Fig. 3). This suggests that chondrocytes expanded on the G5 surface, which does not have extensive stress fibers, also promoting the expression of collagen type II.

Since the present study showed that the levels of chondrogenic genes in the chondrocytes passaged on G5 surface were higher than those in cells passaged on the PS surface, it was interesting to compare the chondrogenic activity in the cell sheets constructed from the chondrocytes, which were previously passaged on either a G5 surface or PS surface. The results showed that the passaged chondrocytes from both surfaces formed intact chondrocyte sheets on a temperature-responsive surface and exhibited a completed cell sheet with the production of cartilage specific genes (Fig. 4). During cell sheet manipulation, all of the chondrocyte sheets appeared to be shrunken (at approximately 50% shrinkage) and dense with honeycomb-like appearance similar to the structure of stress fibers, indicating the reorganization of actin cytoskeleton from a long linear structure to a short structure. These results are consistent with our previous report, which showed that chondrocyte sheets exhibited positive staining of collagen type II with less-intense staining for stress fibers inside the cells [36].

In this study, after layering as a triple-layered sheet, their ECM underneath the cell sheet and adhesion proteins facilitated good adhesiveness of layered chondrocyte sheets, as each of the cell sheets rapidly attached to a new tissue culture surface (Fig. 4a). These results suggest that the dense chondrocyte sheets with their ECM and the reorganization of actin cytoskeleton limited the chondrocytes into a round-shaped cell, leading to the reproduction of chondrogenic-specific genes. This suggestion also remains consistent with the round-shaped cells passaged on G5 surface, which reproduced chondrogenic markers due to the reorganization of stress fibers, as mentioned previously. Moreover, cytoskeletal reorganization in the chondrocytes on both G5 and PS surfaces noticeably influenced the expression of chondrogenic markers in the cell sheets, as the staining of collagen type II and aggrecan were found in all the chondrocyte sheets, particularly in the chondrocyte sheets constructed from the cells on the G5 surface (Fig. 5). Compared to chondrocyte sheets constructed from the cells on PS surface, the dedifferentiation marker did not drastically increase in the chondrocyte sheets constructed from the cells on the G5 surface (Fig. 4b). These results indicate that the prevention of dedifferentiated chondrocytes during cell expansion while retaining chondrogenic activities on the G5 surface is valuable for research in cartilage-like tissue engineering.

## Conclusions

In this study, the effect of cell morphology and cellular aggregation on human articular chondrocytes concerning the maintenance of chondrogenic phenotype was examined using the G5 surface. Chondrogenic phenotype was predominantly maintained in the cells, which formed aggregates and exhibited less production of stress fibers on the G5 surface. This study highlights that a one-step process for cell expansion and maintenance of chondrogenic activities was obtained when the chondrocytes were passaged on the G5 surface, as evidenced by proliferative ability and the expression of chondrogenic markers. Even though the tendency of dedifferentiation marker was increased after serial passages, its expression in the passaged chondrocytes cultured on the G5 surface was lower than that in the chondrocytes on the PS surface. Moreover, the chondrocytes previously cultured on the G5 surface were able to form cell sheets with higher chondrogenic markers and lower dedifferentiation marker compared with other cell sheets constructed from the P2 cells on the PS surface. This study demonstrated that a one-step process for chondrocyte cultivation on the G5 surface could significantly improve chondrogenic activities. Furthermore, the cell sheets constructed from these chondrocytes exhibited high chondrogenic potential and could be beneficial for future research studies concerning the treatment of cartilage damage.

## Additional file

Additional file 1: Schematic illustrations showing the possible signals involved in the one-step process of expansion and differentiation for human chondrocytes on either the G5 or PS surface for chondrocyte sheet construction. (TIFF 20776 kb)
