# Supplementary figures and images for: Maintenance of human chondrogenic phenotype on a dendrimer-immobilized surface for an application of cell sheet engineering
Source: BMC Biotechnol. 2018 Mar 14;18:14. doi: 10.1186/s12896-018-0426-1 (PMC5853058; doi:10.1186/s12896-018-0426-1)

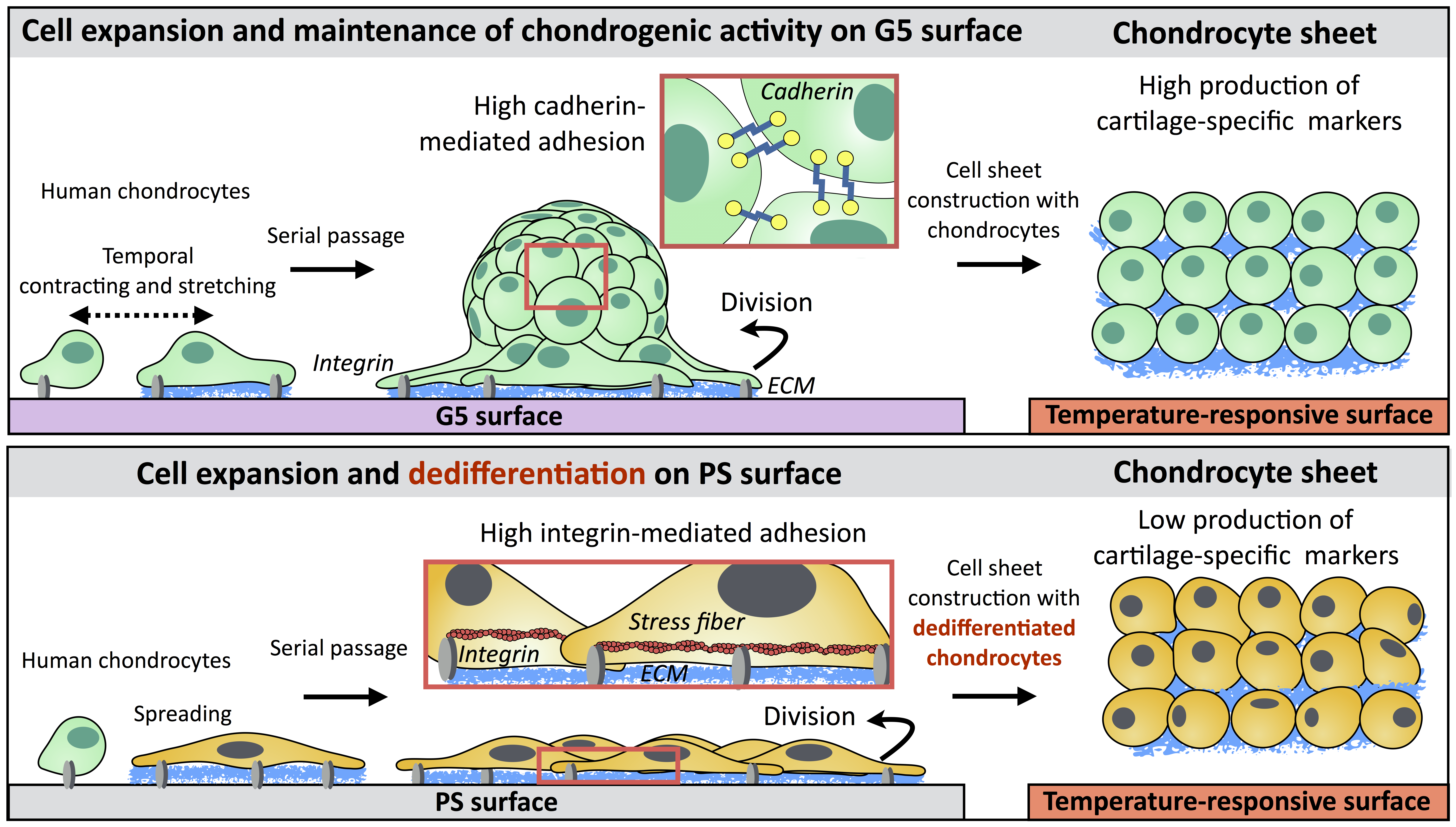

Supplement: Supplementary file 1 — Schematic illustrations showing the possible signals involved in the one-step process of expansion and differentiation for human chondrocytes on either the G5 or PS surface for chondrocyte sheet construction. (TIFF 20776 kb) [file 12896_2018_426_MOESM1_ESM.tif]
